# IL-33/ST2 signalling and crosstalk with FcεRI and TLR4 is targeted by the parasitic worm product, ES-62

**DOI:** 10.1038/s41598-018-22716-9

**Published:** 2018-03-14

**Authors:** Dimity H. Ball, Lamyaa Al-Riyami, William Harnett, Margaret M. Harnett

**Affiliations:** 1 Institute of Infection, Immunity and Inflammation, College of Medical, Veterinary and Life Sciences, University of Glasgow, Glasgow, G12 8TA Scotland; 2 Strathclyde Institute of Pharmacy and Biomedical Sciences, University of Strathclyde, Glasgow, G4 0RE Scotland

## Abstract

ES-62 is a secreted parasitic worm-derived immunomodulator that exhibits therapeutic potential in allergy by downregulating aberrant MyD88 signalling to normalise the inflammatory phenotype and mast cell responses. IL-33 plays an important role in driving mast cell responses and promoting type-2 allergic inflammation, particularly with respect to asthma, via MyD88-integrated crosstalk amongst the IL-33 receptor (ST2), TLR4 and FcεRI. We have now investigated whether ES-62 targets this pathogenic network by subverting ST2-signalling, specifically by characterising how the functional outcomes of crosstalk amongst ST2, TLR4 and FcεRI are modulated by the worm product in wild type and ST2-deficient mast cells. This analysis showed that whilst ES-62 inhibits IL-33/ST2 signalling, the precise functional modulation observed varies with receptor usage and/or mast cell phenotype. Thus, whilst ES-62’s harnessing of the capacity of ST2 to sequester MyD88 appears sufficient to mediate its inhibitory effects in peritoneal-derived serosal mast cells, downregulation of MyD88 expression appears to be required to dampen the higher levels of cytokine production typically released by bone marrow-derived mucosal mast cells.

## Introduction

Reflecting the broad expression of its receptor (IL-33R, comprising ST2 [also known as *IL1RL1*, *IL-1R4*] and IL-1RAcP [*IL-1R3*]) on immune system cells (mast cells and Th2 cells, activated Th1, Treg, ILC2, CD8^+^ T cells and NK cells), IL-33 has been proposed to play key roles in innate and adaptive immunity and also in the resolution of inflammation and promotion of tissue repair^1^. In addition, IL-33 has increasingly been implicated in the pathogenesis of a wide range of inflammatory diseases, but particularly in asthma where GWAS studies have demonstrated clear association of the *IL-33* and *IL-1RL1* genes with human asthma and ST2-deficiency in mice results in suppression of acute eosinophilic inflammation and airway hyper-responsiveness (AHR)^1,2^. Mast cells are a major player in the induction and promulgation of such allergic inflammation as allergen-induced crosslinking of FcεRI results in the release of a wide range of pro-inflammatory mediators (including histamine, cytokines, proteases and prostaglandins), which regulate a variety of disease parameters including recruitment of inflammatory cells, smooth muscle contraction and vascular permeability. Moreover, these FcεRI-mediated mast cell responses can be modulated by various stimuli including LPS (via TLR4) and IL-33, both of which have been shown to amplify mast cell responsiveness and exacerbate AHR^3–8^ and perhaps pertinently, are present during bacterial infection, a clinical situation that can aggravate established asthma^9^.

By contrast, ES-62, a phosphorylcholine-containing glycoprotein immunomodulator secreted by the parasitic worm, *Acanthocheilonema viteae*, protects against airway inflammation and lung pathology in both acute and chronic models of asthma^10–12^ by subverting TLR4 signalling and inducing mast cell hyporesponsiveness^10,12–14^. In particular, ES-62 appears to act by targeting aberrant signalling via MyD88 to reset the homeostatic regulatory: effector cell balance of the immune response and thereby normalise dysregulated inflammatory phenotypes in allergic and autoimmune disease^15,16^. MyD88 has recently been suggested to provide a key regulatory node in the convergent crosstalk amongst IL-33/ST2, LPS/TLR4 and FcεRI that leads to the generation of type-2 responses^1,5,17–22^. This idea is consistent with the ability of ST2 to block MyD88’s association with TLR4^23^ and in macrophages, inhibit LPS/TLR4-mediated NF-κB activation and consequent release of IL-6 and IL-12, cytokines that promote Th1/Th17-responses, by sequestering MyD88 via its TIR domain. As LPS signalling upregulates ST2 expression in monocytes/macrophages, these data led to the proposal that ST2 may act to limit LPS/TLR4 proinflammatory responses, a hypothesis supported by the failure of ST2-deficient mice to develop endotoxin tolerance^1,24^.

Thus, to explore the possibility that ES-62 may act to suppress this pathogenic network in allergic conditions, we have now investigated whether it can subvert IL-33/ST2/MyD88 signalling to modulate the proinflammatory responses resulting from crosstalk between ST2, FcεRI and TLR4. Specifically, by exploiting ST2-deficient cells, we first characterised how IL-33/ST2 signalling differentially shapes FcεRI- and TLR4-driven responses in distinct subsets of mast cells, before moving on to determine the ST2-dependence of ES-62-driven modulation of this pathogenic network. This approach showed that ES-62 could suppress IL-33/ST2-induced cytokine (IL-6 and IL-13) responses in both serosal peritoneal-derived mast cells (PDMCs) and bone marrow-derived mucosal mast cells (BMMCs). However, whilst the ability of ES-62 to mediate its inhibitory effects on LPS/TLR4 or IL-33/ST2 + LPS/TLR4 responses was found to be ST2-dependent in PDMCs, this was not the case in BMMCs. Collectively, therefore, our data support the utility of exploiting ES-62 to dissect the mechanisms regulating inflammatory networks and the potential of MyD88 as a target for therapeutic intervention in the differential (receptor-, inflammation site- and subtype-specific-) pathogenic mast cell responses associated with allergy.

## Results

### IL-33 signalling and pro-inflammatory responses in PDMCs and BMMCs

**Figure 1:**
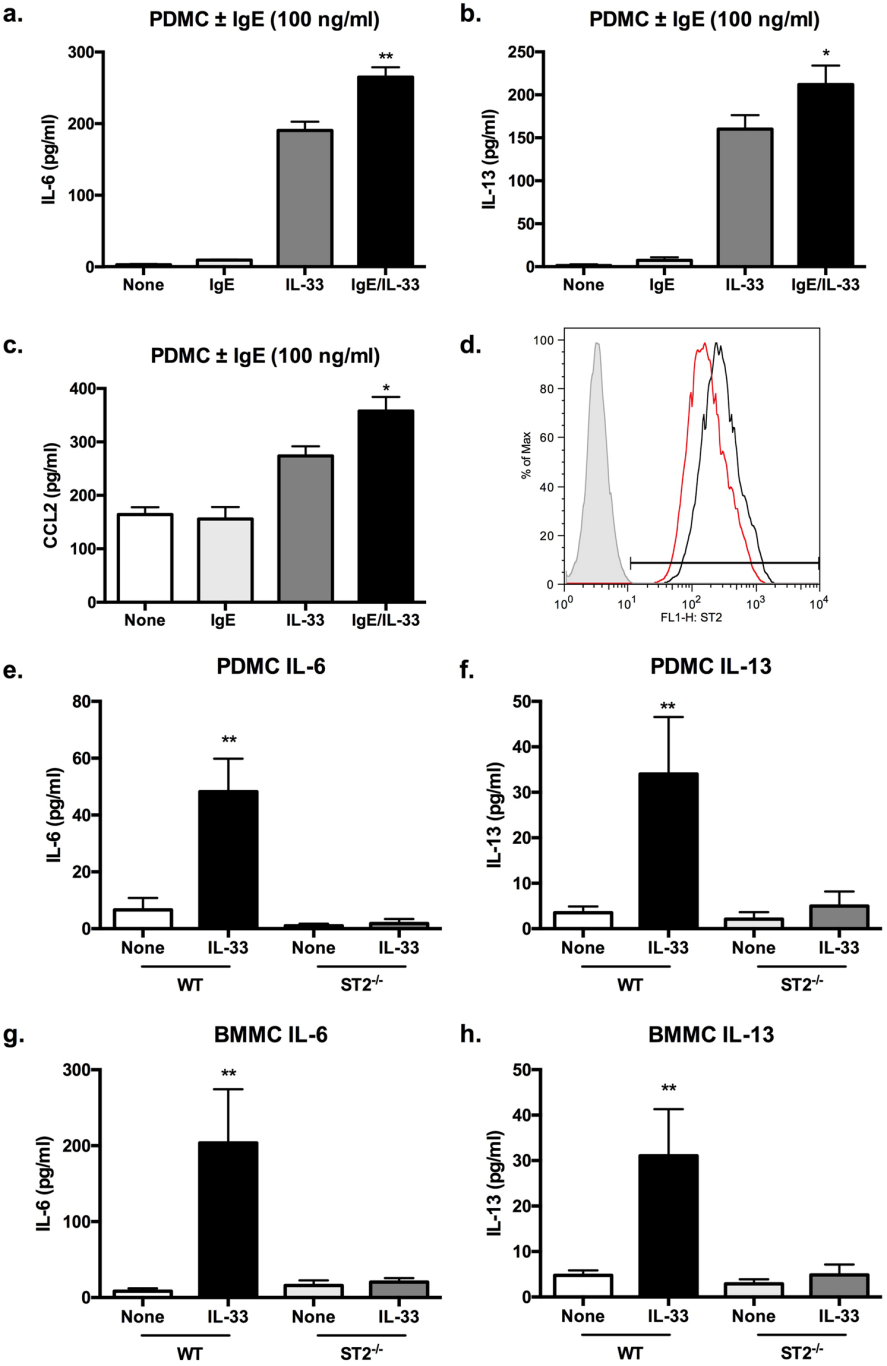
IL-33 stimulated cytokine production is ST2-dependent and enhanced by IgE-mediated sensitisation of PDMCs and BMMCs. PDMCs (**a**–**f**) and BMMCs (**g**,**h**) derived from WT or ST2^−/−^ (BALB/c) mice were sensitized (**a**–**h**) or not (**a**–d**)** with murine anti-DNP IgE (0.5 μg/ml) overnight then stimulated with IL-33 (100 ng/ml, (**a**–**c**); 10 ng/ml, e-h) for 24 h at 37 °C before release of IL-6, IL-13 or CCL2 was measured by ELISA. Data are presented as the mean values of triplicate samples ± SD and are from single experiments representative of 2 (**a**–**c**) and 3 (**e**–**h**) independent experiments. Statistical analysis was performed using One-way ANOVA with Bonferroni’s multiple comparison test where *p < 0.05 and **p < 0.01. Flow cytometric analysis (**d**) of PDMCs sensitized (black line), or not (red line), with murine anti-DNP IgE overnight were gated for live 7AAD^-^ cells and then analysed for ST2 expression against the relevant isotype control obtained with non-sensitised cells (grey plot).

**Figure 2:**
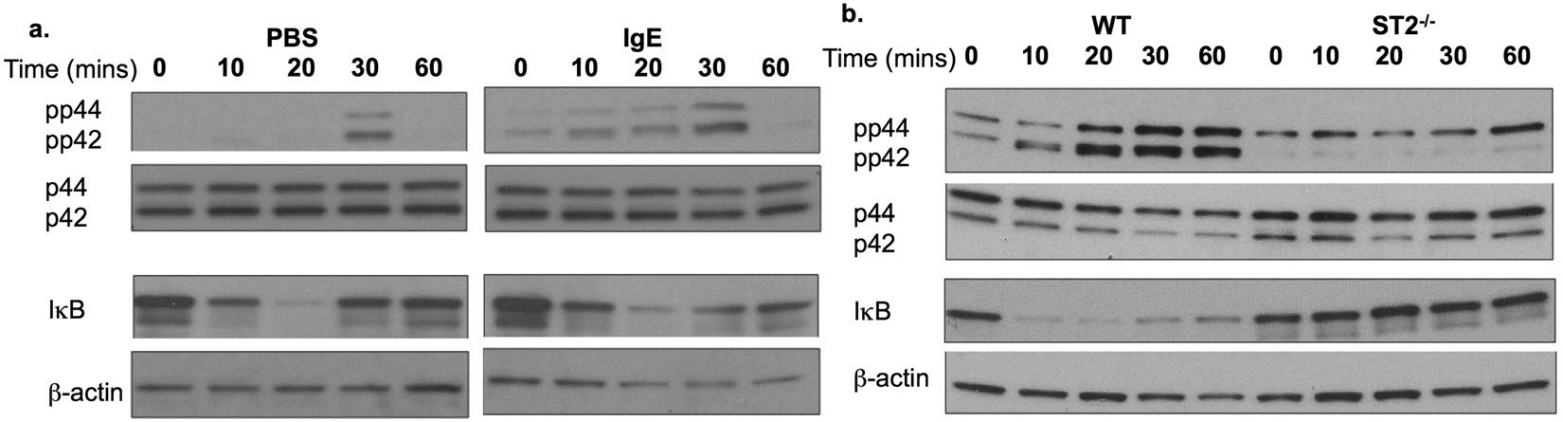
IL-33-stimulated ERK and NF-κB signalling is ST2-dependent and enhanced by IgE-mediated sensitisation of PDMCs and BMMCs. (**a**) PDMCs sensitized with murine anti-DNP IgE (0.5 μg/ml) overnight (IgE) or not (PBS) were cultured with IL-33 (10 ng/ml) for the indicated times and expression of activated (dually phosphorylated) and total ERK1 (p44) and ERK2 (p42) MAPK and IκBα analysed by Western blotting. β-actin was used as a loading control. (**b**) Wild type (WT) and ST2 ^−/−^ BMMCs sensitised with IgE were stimulated with IL-33 (10 ng/ml) for the indicated times and expression of activated (dually phosphorylated) and total ERK1 (p44) and ERK2 (p42) MAPK and IκBα analysed by Western blotting. β-actin was used as a loading control.

IL-33 stimulates production of the cytokines, IL-6 and IL-13 and the chemokine, CCL2 by PDMCs and BMMCs: the release of all three pro-inflammatory mediators is enhanced by the sensitisation of mast cells by DNP-specific IgE and this is accompanied by the induction of a slight increase in expression of the IL-33 receptor, ST2 (Fig. 1a–d and results not shown). Supporting the role of IgE-mediated upregulation of ST2 in the enhancement of IL-33 functional responses, the release of lL-6 and IL-13 by PDMCs and BMMCs in response to stimulation with IL-33 + IgE is completely dependent on ST2-expression (Fig. 1e–h). Moreover, whilst IgE sensitisation of mast cells is associated with increased IL-33-mediated activation of ERK and NF-κB activation (the latter assessed in terms of degradation of the inhibitory element IκB), such IL-33 signalling was essentially abrogated in ST2-deficient PDMC and BMMC populations (Fig. 2a,b and results not shown). Enhancement of these key IL-33/ST2 signals^1^ therefore provides a molecular rationale for the increased mast cell responses to IL-33 observed following sensitisation with IgE.

**Figure 3:**
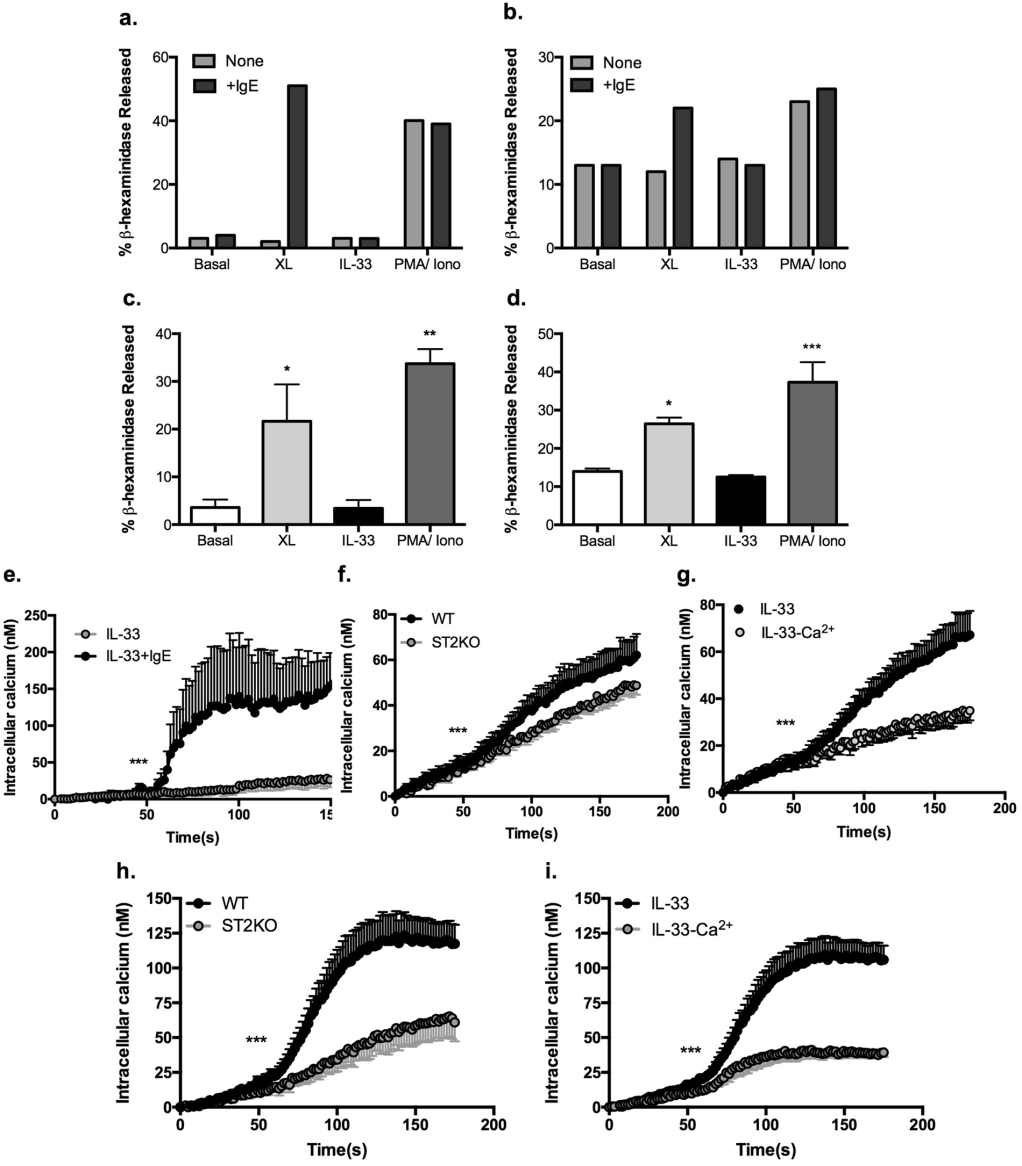
The failure of IL-33 to induce mast cell degranulation is associated with lack of intracellular mobilisation of calcium. PDMCs (**a**,**c**) and BMMCs (**b**,**d**) were sensitized with murine anti-DNP IgE (0.5 μg/ml) or not (**a**,**b**) and then stimulated with DNP-HSA (0.5 µg/ml) to induce cross-linking of FcεRI (XL), IL-33 (10 ng/ml) or PMA plus Ionomycin (both 1 μM) for 30 mins at 37 °C. Degranulation was determined as the % β-hexosaminidase released relative to the total activity of the cells and data are presented from a single experiment (**a**,**b**) or mean ± SEM values from the combined results of five (**c**) or three (d; IL-33, n = 2) independent experiments where statistical analysis was by one-way ANOVA with Bonferroni’s post test and *p < 0.05, **p < 0.01 and ***p < 0.001. WT or ST2 ^−/−^ PDMCs (**e**–**g**) and BMMCs (**h**,**i**) loaded with Fura-2/AM and sensitised or not (**e**) with murine anti-DNP IgE (0.5 μg/ml) were stimulated at 50 s in serum-free HBSS with 100 ng/ml IL-33. Calcium mobilisation was recorded in real-time and for the analysis of intracellular mobilisation alone, the cells were stimulated in calcium-free buffer supplemented with 100 µM EGTA to remove all extracellular calcium (−Ca^2+^; g, i). Data are presented as the mean (baseline subtracted) values ± SEM from replicate biological analyses collated from at least two independent experiments as follows: for PDMCs, e: IL-33, n = 3, IL-33 + IgE, n = 4; f: WT n = 6, ST2^−/−^ n = 5; g: IL-33 n = 7, IL-33-Ca^2+^ n = 7; for BMMCs, h: WT n = 6, ST2^−/−^ n = 5; i: IL-33 n = 10, IL-33-Ca^2+^ n = 9. Statistical analysis was by two-way ANOVA where ***p < 0.001.

By contrast, despite undergoing degranulation in response to antigen (DNP-HSA)-induced crosslinking of FcεRI (XL) or stimulation with PMA plus ionomycin (PMA/Iono), neither mast cell subtype degranulates in response to IL-33, either in the presence or absence of IgE sensitisation (Fig. 3a–d). As strong mobilisation of intracellular and extracellular calcium is key to FcεRI-mediated mast cell degranulation^25,26^, we therefore investigated whether this reflected an inability of IL-33 to induce such signalling. Prior sensitisation by IgE was found to license ST2-dependent, IL-33 mobilisation of calcium (Fig. 3e–i): however, depletion of extracellular calcium by EGTA revealed that the low levels of calcium signalling observed, predominantly reflected influx of extracellular calcium with little or no release from intracellular stores (Fig. 3g,i), findings consistent with the failure of IL-33 to cause degranulation.

### Cross talk between ST2 and FcεRI signalling

**Figure 4:**
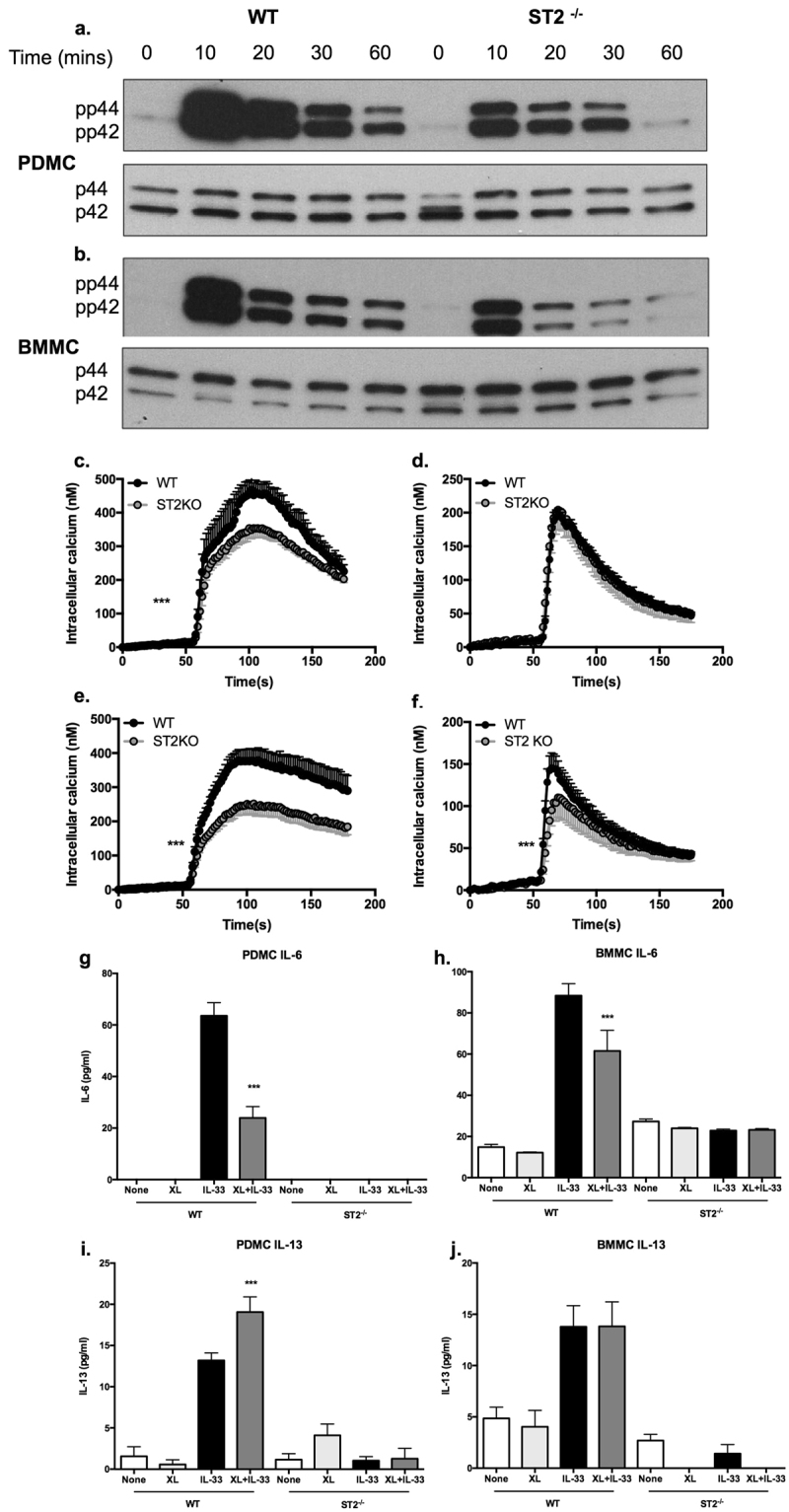
FcεRI signalling and functional responses are modulated by ST2 expression. WT or ST2^−/−^ PDMCs (**a**) or BMMCs (**b**) sensitized with murine anti-DNP IgE (0.5 μg/ml) overnight were cultured with DNP-HSA (0.5 μg/ml) to induce cross-linking (XL) of FcεRI for the indicated times and expression of pp44/pp42 dually phosphorylated and activated ERK1 and ERK2 relative to their total expression was analysed by Western blotting. WT or ST2^−/−^ PDMCs (**c**,**d**) and BMMCs (**e**,**f**) sensitised with murine anti-DNP IgE (0.5 μg/ml) and loaded with Fura-2/AM were stimulated at 50 s in serum-free HBSS with DNP-HSA (0.5 μg/ml) to induce cross-linking (XL) of FcεRI. Intracellular calcium mobilisation and influx were recorded in real-time, where for the analysis of intracellular mobilisation alone (**d**,**f**), the cells were stimulated in calcium-free buffer supplemented with 100 µM EGTA to remove all extracellular calcium. Data are presented as the mean (baseline subtracted) values ± SEM from replicate biological analyses from at least two independent experiments as follows: for PDMCs, (**c** and **d**) WT n = 6, ST2^−/−^ n = 6; for BMMCs, (**e)** WT n = 7, ST2^−/−^ n = 8; (**f)** WT n = 9, ST2^−/−^ n = 10. Statistical analysis was by two-way ANOVA where ***p < 0.001. PDMCs (**g**,**i**) and BMMCs (**h**,**j**) derived from WT or ST2^−/−^ mice were sensitized with murine anti-DNP IgE (0.5 μg/ml) overnight and stimulated with IL-33 (10 ng/ml) and/or DNP-HSA (0.5 µg/ml) for 24 h at 37 °C before release of IL-6 (**g**,**h**) and IL-13 (**i**,**j**) was measured by ELISA. Data are presented as the mean values of triplicate samples ± SD from single experiments representative of at least 2 independent experiments. Statistical analysis is by one-way ANOVA and Bonferroni’s post test where ***p < 0.001.

Previous reports had suggested acute IL-33 and FcεRI signalling to be synergistic in promoting mast cell cytokine production^27–30^: perhaps reflecting this, FcεRI-mediated ERK activation and calcium mobilisation were found to be reduced in ST2-deficient PDMCs and BMMCs (Fig. 4a–f). However, and consistent with our data that IL-33/ST2 calcium signalling predominantly reflects influx (Fig. 3g,i), ST2-deficiency had no effect on the strong mobilisation of intracellular calcium stores (evident in the absence of extracellular calcium resulting from incubation with EGTA) observed following FcεRI crosslinking in PDMCs and only partially (~30%) inhibited this in BMMCs (Fig. 4d,f). Although, as shown in our previous studies^13,14^, we found crosslinking of FcεRI to induce variable levels of cytokine release by PDMCs (generally very low) and BMMCs, typically, FcεRI crosslinking resulted in little or no release of IL-6 or IL-13 from either mast cell subtype derived from our WT and ST2^−/−^ (BALB/c) littermate mice. Interestingly, therefore costimulation of such WT PDMCs or BMMCs via ST2 (with IL-33) and FcεRI resulted in a reduced production of IL-6 relative to that observed with IL-33 alone (Fig. 4g,h). By contrast, IL-13 release was not suppressed and indeed, such co-stimulation resulted in an ST2-dependent increase in production of IL-13 by PDMCs and whilst levels were generally not substantially altered, similarly, a significant increase was found in 1 of the 3 BMMC experiments performed (Fig. 4i,j and results not shown). Thus, these data perhaps suggest that cross talk between FcεRI and IL-33/ST2 may not simply enhance antigen-specific responses but rather, depending on the mast cell subtype and inflammatory context, modulate the phenotype of response generated.

### Cross talk between ST2 and TLR4 signalling

**Figure 5:**
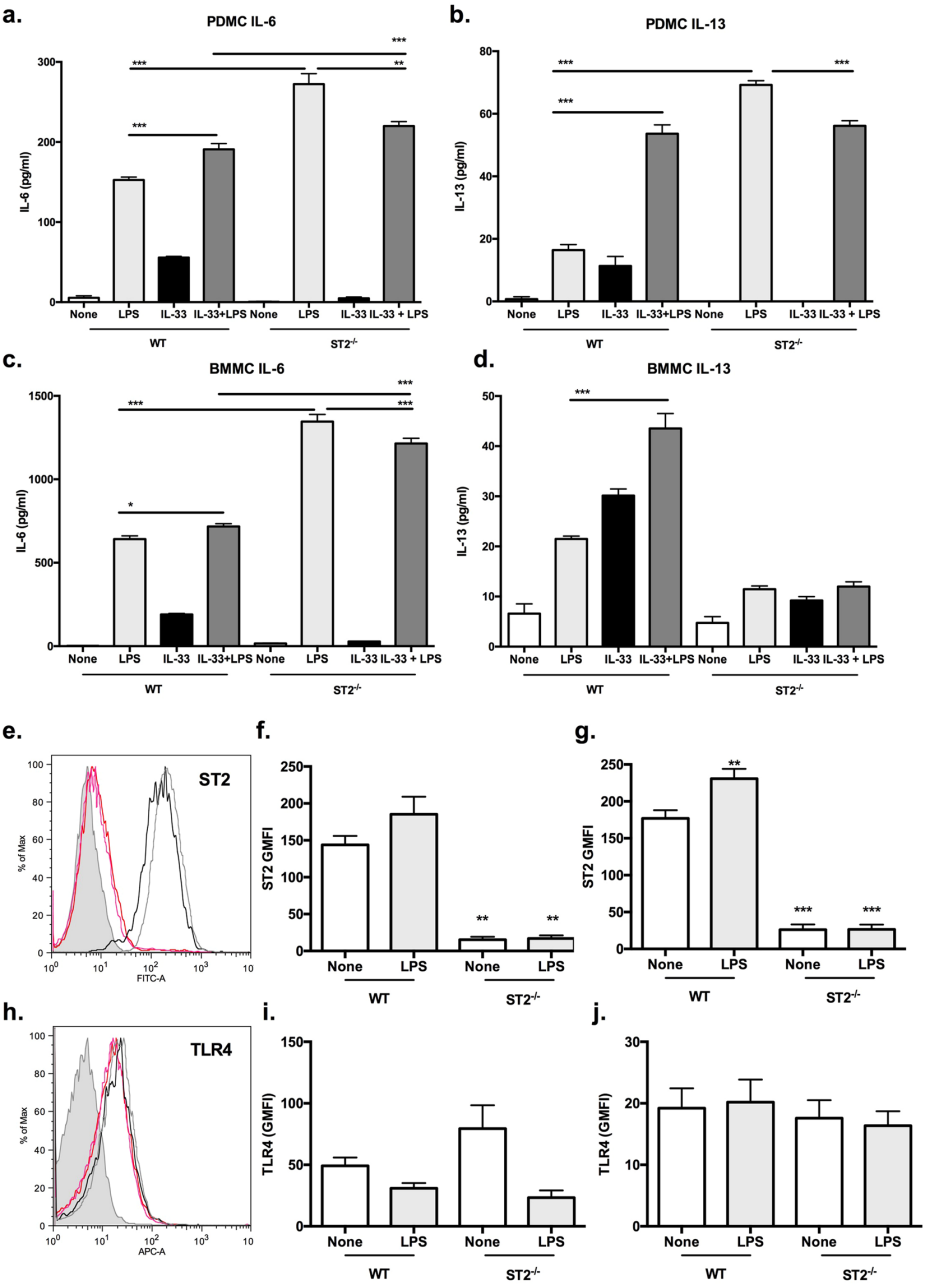
PDMCs and BMMCs exhibit differential TLR4-ST2 cross-talk. PDMCs (**a**,**b**) and BMMCs (**c**,**d**) derived from WT or ST2^−/−^ mice were sensitized with murine anti-DNP IgE (0.5 μg/ml) overnight and then stimulated with LPS (0.5 µg/ml) and/or IL-33 (10 ng/ml) for 24 h at 37 °C before release of IL-6 (**a**,**c**) and IL-13 (**b**,**d**) was measured by ELISA. Data are presented as the mean values of triplicate samples ± SD from single experiments representative of three independent experiments. Statistical analysis is by one-way ANOVA and Bonferroni’s post test where *p < 0.05, **p < 0.001 and ***p < 0.001. Flow cytometric analysis (**e**–**j**) of WT and ST2^−/−^ PDMCs (**f**,**i**) or BMMCs (**e**,**g**,**h**,**j**) sensitized with murine anti-DNP IgE and treated with LPS (0.5 μg/ml; WT, grey line; ST2^−/−^, pink line) or not (WT, black line; ST2^−/−^, red line) overnight. Live (7AAD^−^) CD117^+^FcεRI^+^ cells were analysed for ST2 (**e**–**g**) and TLR4 (**h**–**j**) expression relative to the relevant isotype controls (grey plots) in terms of the geometric mean fluorescence (GMFI). Data presented are the mean ± SEM values combined from at least three independent experiments and statistical analysis is by one-way ANOVA and Bonferroni’s post-test where **p < 0.01 and ***p < 0.001 relative to the “None” WT control.

ST2 has previously been reported to limit LPS/TLR4 and BLP/TLR2 pro-inflammatory responses in monocytes^21,22^, putatively via sequestration of MyD88^24^. As we have previously demonstrated LPS also to mobilise calcium influx and induce cytokine responses in PDMCs and BMMCs^13^, we therefore investigated the effects of ST2 signalling on LPS/TLR4 responses in such mast cells (Fig. 5a–d). Using ST2-deficient cells, this revealed that ST2 similarly acted to limit LPS-induced IL-6 release from both PDMCs (1.40 ± 0.20 fold release in ST2^−/−^ relative to WT cells, n = 3 experiments) and BMMCs (1.75 ± 0.22 fold release in ST2^−/−^ relative to WT cells, n = 3 experiments) as their secretion of this cytokine was substantially enhanced in its absence (Fig. 5a,c). By contrast, whilst ST2 deficiency strongly increased LPS-induced release of IL-13 from PDMCs (5.36 ± 0.52 fold release in ST2^−/−^ cells, n = 3 experiments), it surprisingly abrogated all release of this cytokine by BMMCs (Fig. 5b,d). However, and reflecting the contradictory and divergent effects of ST2 signalling on LPS responses reported in studies exploiting IL-33, ST2-deficient cells, (decoy) fusion proteins and neutralising antibodies^1,22,24,31,32^, we find that IL-33 signalling significantly enhances LPS-induction of IL-6 (PDMCs: 1.19 ± 0.06; BMMCs: 1.22 ± 0.14 fold release relative to LPS alone, n = 3 experiments) and particularly, IL-13 (PDMCs: 3.60 ± 0.58; BMMCs: 2.24 ± 0.36 fold release relative to LPS alone, n = 3 experiments) in both mast cell subtypes (Fig. 5a–d). Moreover, this IL-13 enhancement is mediated via ST2 as it is lost in ST2-deficient mast cells with in some cases, IL-33 + LPS responses being less than those seen in response to (deregulated) LPS signalling alone: the slightly higher levels of IL-6 (but not IL-13) elicited by IL-33 + LPS in ST2^−/−^ relative to WT mast cells presumably simply reflect the loss of the ST2-brake on LPS (alone) signalling.

**Figure 6:**
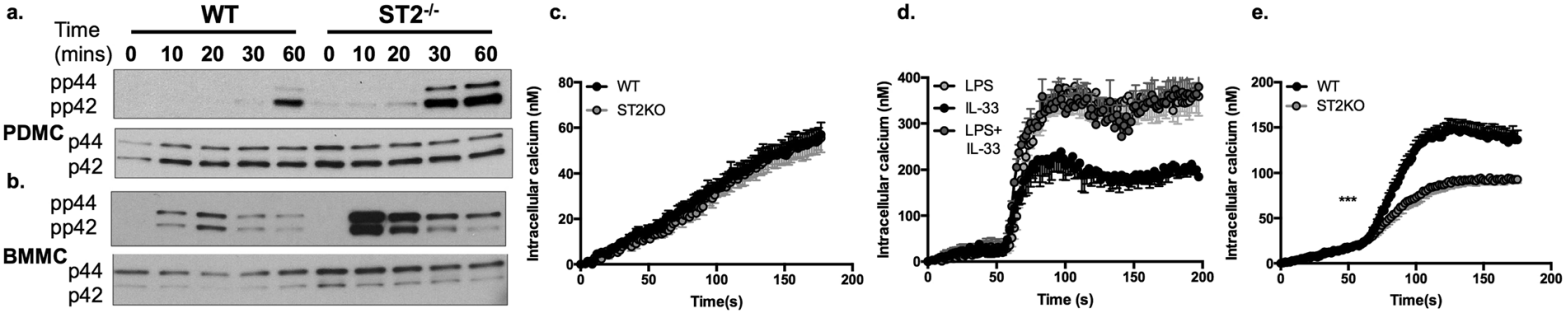
PDMCs and BMMCs exhibit differential ST2-dependence for calcium signalling. WT or ST2^−/−^ PDMCs (**a**) or BMMCs (**b**) sensitized with murine anti-DNP IgE (0.5 μg/ml) overnight were cultured with LPS (0.5 μg/ml) for the indicated times and expression of pp44/pp42 dually phosphorylated and activated ERK1 and ERK2 relative to their total expression was analysed by Western blotting. PDMCs (**c**,**d**) and BMMCs (**e**) sensitised with murine anti-DNP IgE (0.5 μg/ml) and loaded with Fura-2/AM were stimulated at 50 s in serum-free HBSS with LPS (0.5 μg/ml) and/or IL-33 (10 ng/ml). Calcium mobilisation data are presented as the mean (baseline subtracted) values ± SEM from replicate biological analyses as follows: for PDMCs, (**c)** WT n = 6, ST2^−/−^ n = 6; (**d)** LPS n = 3, IL-33 n = 3, LPS + IL-33 n = 3, for BMMCs, (**e)** WT n = 9, ST2^−/−^ n = 9. Statistical analysis was by two-way ANOVA where ***p < 0.001.

To address the mechanisms underpinning the differential outcomes of IL-33/ST2-dependent modulation of LPS responses in PDMCs and BMMCs, we assessed the effects of LPS on ST2 and TLR4 expression in WT and ST2-deficient mast cells. This showed that LPS slightly upregulated expression of ST2, but not TLR4, and that TLR4 expression was not significantly modulated in ST2-deficient cells (Fig. 5e–j). Consistent with the enhancement of cytokine responses observed, LPS-stimulated ERK activation was strongly enhanced in both PDMCs and BMMCs from ST2-deficient mice (Fig. 6a,b). Interestingly, therefore, whilst ST2-mediated deficiency or IL-33 did not impact on LPS-mediated calcium mobilisation in PDMCs (Fig. 6c,d), such signalling was substantially reduced in ST2^−/−^ BMMCs (Fig. 6e), demonstrating a rather unexpected dependence on ST2 expression for such signalling in this mucosal-like mast cell subtype.

### ES-62 targets ST2-TLR4 crosstalk for mast cell immunomodulation

**Figure 7:**
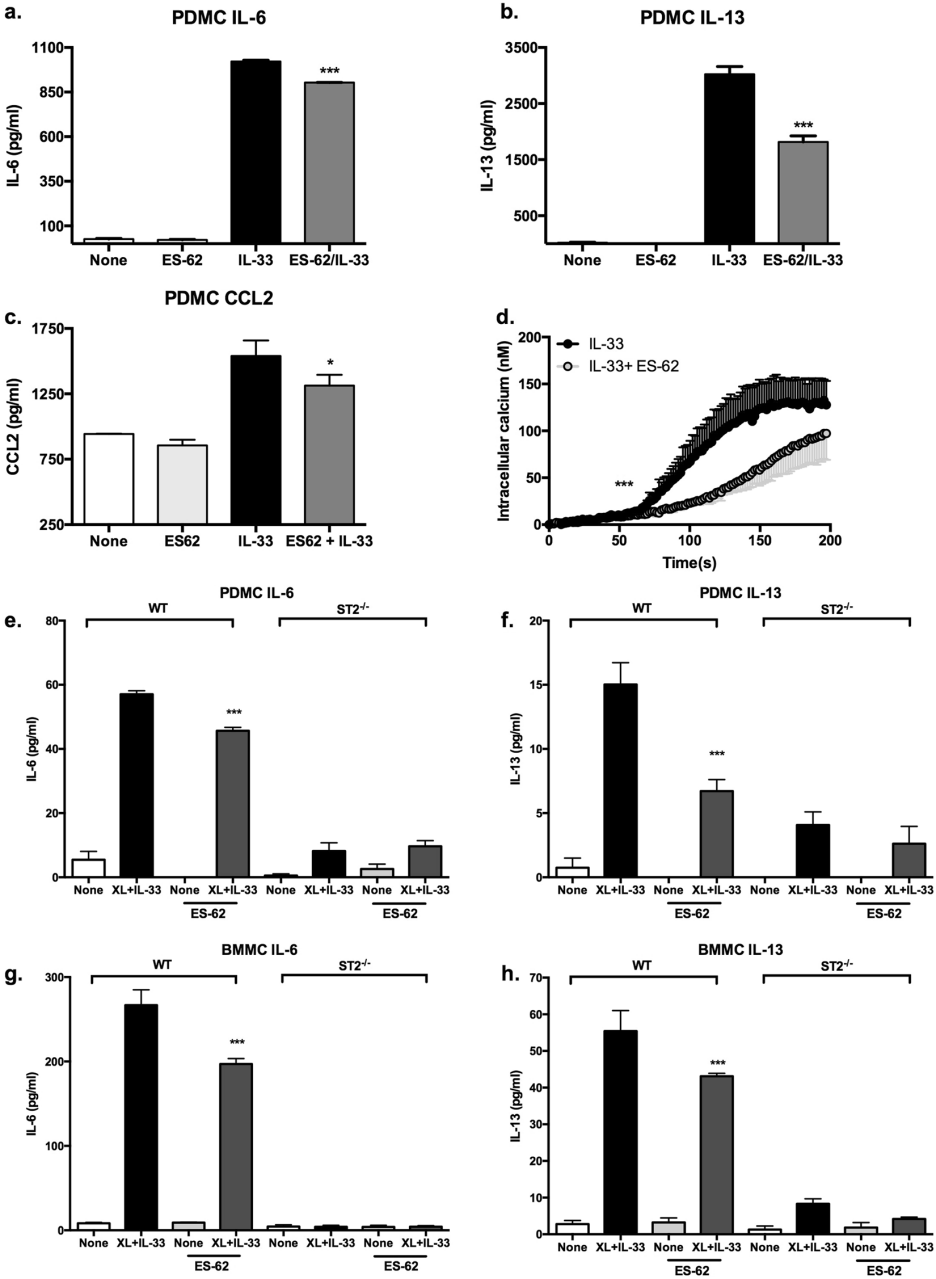
ES-62 modulates IL-33 responses. PDMCs were sensitized with murine anti-DNP IgE (0.5 μg/ml) in the presence or absence of ES-62 (2 µg/ml) overnight and then incubated in medium ± IL-33 (10 ng/ml) for 24 h at 37 °C before release of IL-6, IL-13 and CCL2 was measured by ELISA (**a–c**). Data are mean ± SD values where ***p < 0.001 and are from single experiments, which for IL-6 and IL-13 were representative of four-six experiments. Analysis of the latter data shows that ES-62 significantly reduces IL-33-induced IL-6 and IL-13 production by PDMCs to 66.03 ± 16.82 n = 5, p < 0.05 and 59.85 ± 14 n = 6, p < 0.01 of the control responses respectively and by BMMCs to 81.23 ± 5.72 n = 4, p < 0.05 and 75.75 ± 8.13 n = 5, p < 0.05 of the control responses respectively. PDMCs (**d**) were sensitised with murine anti-DNP IgE (0.5 μg/ml) in the presence or absence of ES-62 (2 µg/ml) and then loaded with Fura-2/AM and stimulated at 50 s in serum-free HBSS with IL-33 (10 ng/ml). Calcium mobilisation data are presented as the mean (baseline subtracted) values ± SEM where for IL-33, n = 3 and for IL-33 + ES-62, n = 4 replicate biological samples from a single experiment representative of at least three independent experiments. Statistical analysis was by two-way ANOVA where *** p < 0.001. WT and ST2^−/−^ PDMCs (**e**,**f**) and BMMCs (**g**,**h**) were sensitized with murine anti-DNP IgE (0.5 μg/ml) in the presence or absence of ES-62 (2 µg/ml) overnight and then incubated in medium ± 0.5 µg/ml DNP-HSA plus 10 ng/ml IL-33 (XL + IL-33) for 24 h at 37 °C before release of IL-6 (**e**,**g**) and IL-13 (**f**,**h**) was measured by ELISA. Data are mean ± SD values where ***p < 0.001. Analysis of 3 independent experiments showed that ES-62 reduced IL-33 + XL-stimulated IL-6 and IL-13 production by PDMCs to 79.27 ± 12.42 and 59.98 ± 10.07, p < 0.05 of the control responses respectively and by BMMCs to 65.11 ± 26.72 and 65.60 ± 20.43, p < 0.05 of the control responses in WT, but not ST2KO mast cells.

In direct contrast to the enhanced functional responses observed as a result of crosstalk between IL-33/ST2 and LPS/TLR4 signalling (Figs 5 and 6), but consistent with its ability to subvert TLR4 signalling to an anti-inflammatory phenotype, ES-62 significantly reduces IL-33-induced IL-6, IL-13 and CCL2 production by PDMCs (Fig. 7a–c). This inhibition does not reflect modulation of ST2 expression^13^ but it is associated with reduced IL-33-mediated calcium mobilisation in these cells (Fig. 7d): these findings are reminiscent of our previous data demonstrating that whilst LPS/TLR4 crosstalk with FcεRI results in increased calcium mobilisation and cytokine release, ES-62/TLR4 signalling inhibits FcεRI- and LPS-mediated calcium mobilisation and functional responses in mast cells^10,13^. As ST2 cross-talks with both FcεRI and TLR4, and indeed FcεRI and TLR4 show dependence on ST2 for calcium mobilisation, we therefore next investigated whether any inhibitory effects of ES-62 were impacted by ST2 expression.

**Figure 8:**
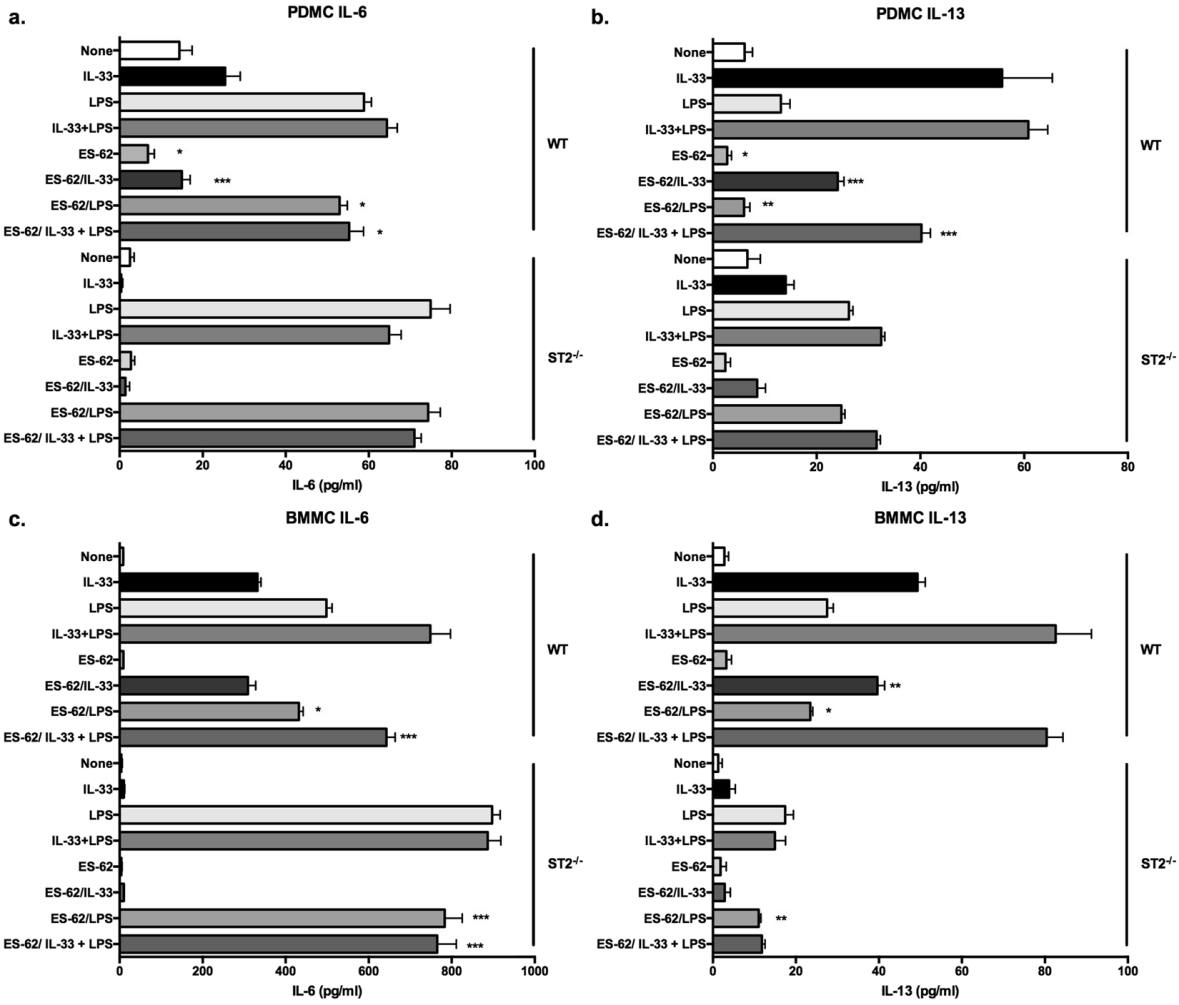
ST2 deficiency impacts differentially on ES-62-mediated modulation of signalling in PDMCs and BMMCs. WT and ST2^−/−^ PDMCs (**a**,**b**) and BMMCs (**c**,**d**) were sensitized with murine anti-DNP IgE (0.5 μg/ml) in the presence or absence of ES-62 (2 µg/ml) overnight and then incubated in medium ± IL-33 (10 ng/ml) and/or LPS (0.5 µg/ml) for 24 h at 37 °C before release of IL-6 (**a**,**c**) and IL-13 (**b**,**d**) was measured by ELISA. Data are mean ± SD values from a single experiment representative of at least two independent experiments where ***p < 0.001. Statistical analysis where three independent experiments were performed showed that whilst treatment with ES-62 resulted in 39.5 ± 19.8% p < 0.05 of the LPS- and 78.8 ± 9.0% p < 0.05 of IL33 + LPS-stimulated IL-13 production by WT PDMCs relative to the appropriate control responses, this inhibition was abolished (106 ± 10 and 106 ± 6% of the LPS and IL-33 + LPS control responses, respectively) in ST2KO PDMCs. By contrast, the LPS-induced release of IL-13 was inhibited by ES-62 in both WT and ST2^−/−^ BMMCs (70.6 ± 6.7% WT and 63.5 ± 9.2% p < 0.05 control responses, respectively).

ES-62 significantly reduced IL-33 + XL-stimulated IL-6 and IL-13 responses in WT mast cells: however, there were no significant differences between control and ES-62-treated PDMCs or BMMCs in ST2-deficient cells where the functional responses of ST2/FcεRI crosstalk were essentially abrogated (Fig. 7e–h). Likewise, the ES-62 mediated suppression of LPS- and IL-33 + LPS-stimulated release of either IL-6 or IL-13 by PDMCs (Fig. 8a,b) was found to be lost in ST2-deficient cells. By contrast, the inhibition of LPS- and IL-33 + LPS-stimulation of IL-6 production remained intact in ST2^−/−^ BMMCs and likewise, although responses were very low in the ST2-deficient cells, the LPS-induced release of IL-13 was inhibited by ES-62 in WT and ST2^−/−^ BMMCs (Fig. 8c,d).

## Discussion

The filarial nematode-derived immunomodulator, ES-62 protects against development of asthma in both acute and chronic mouse models^11,12^ at least in part by suppressing FεcRI- and LPS/TLR4-mediated activation of mast cells and downregulation of type-2 inflammatory responses^10–14^. IL-33/ST2 signalling has been proposed to fine-tune mast cell responses and indeed, FcεRI signalling has been reported to induce, in a calcium-dependent manner, IL-33 production by mast cells to amplify IgE-mediated inflammation^33^. Moreover, blockade of IL-33/ST2 signalling was found to disrupt the late phase inflammatory response in a passive cutaneous anaphylaxis model of allergen-IgE-driven inflammation^34^. LPS has also been shown to augment FcεRI-driven mast cell responses^3–5,13,35,36^ but in this case, it does not appear to involve the release of IL-33 from these cells^34^. Nevertheless, bacterial-induced tissue damage causes release of IL-33 and LPS signals, via TLR4, to elicit IL-33 production from monocytes and macrophages^37,38^, mucosal epithelial cells^39^ and DCs^40,41^. Thus, as both LPS/TLR4 and IL-33/ST2 signal via MyD88, there is potential for crosstalk between TLR4 and IL-33R (ST2) on mast cells to promote pathogenesis and exacerbation of asthma. Importantly, therefore given the protection afforded by ES-62 in acute and chronic models of asthma, our studies have identified that the differential cytokine responses in serosal (PDMC) and mucosal (BMMC) mast cell subsets shaped by complex interactions of ST2, FcεRI and LPS/TLR4 signals can be modulated by ES-62 via both ST2-dependent and independent mechanisms.

ES-62 acts to subvert inflammatory responses by normalising aberrant MyD88 signalling in a range of cell types (reviewed)^16,42^: thus reports that the augmentation of cytokine responses resulting from costimulation of BMMCs with IL-33 and allergen was abolished in MyD88 (C57BL/6)-deficient mice^27^, provides a rationale for the ability of the parasite product to target such FcεRI/ST2/TLR4 crosstalk in mast cells. Intriguingly therefore, given its ability to target ST2 and/or TLR4 responses in mast cells, ES-62 does not downregulate MyD88 expression in PDMCs^13^: rather, as evidenced by the loss of its ability to modulate TLR4-associated responses in ST2^−/−^ PDMCs, it appears to harness the negative regulatory properties of ST2 in sequestrating MyD88 to limit LPS/TLR4-driven (pathogenic) cytokine responses in macrophages^24^. By contrast, ES-62 can still dampen LPS or IL-33 + LPS functional responses in ST2^−/−^ BMMCs, a finding reminiscent of its ability to inhibit TLR4-driven IL-12 production by monocytes from ST2-deficient mice^43^. These data indicate that ES-62 utilises distinct mechanisms to inhibit LPS/TLR4-associated cytokine responses in PDMCs and BMMCs. Interestingly, therefore, whilst ES-62 downregulates expression of PKCα in both mast cell subtypes, it additionally downregulates MyD88 and TLR4/MyD88-associated PKCs, PKC-δ and PKC-ε in BMMCs but not PDMCs^13,14^. Collectively, therefore, these data suggest that whilst exploiting the ability of ST2 to sequester MyD88 is sufficient to enable ES-62 to uncouple TLR-mediated pro-inflammatory responses in PDMCs, direct degradation of MyD88 and associated transducers is required to reset homeostatic regulation in the context of the stronger TLR-biased cytokine responses of BMMCs.

Indeed, MyD88-integrated crosstalk between LPS/TLR4 and IL-33/ST2 appears to be complex, varying with respect to the mast cell subtype and particular response investigated. Also, the role of ST2 can differ depending on whether the effects of IL-33 stimulation or ST2-deficiency are examined (reviewed^1^), for example as exemplified by the divergent effects we have observed in the case of LPS-mediated stimulation of IL-13 responses in PDMCs and BMMCs. When the effects of IL-33 signalling and ST2-deficiency converge, this may reflect that IL-33 signalling can relieve ST2-sequestration of MyD88 and consequently promote TLR4 signalling and functional responses. This idea may go some way to reconciling the apparently contradictory reports that whilst ST2 KO studies indicate that (in the absence of IL-33) ST2 negatively regulates TLR4 signalling, IL-33 promotes it, the latter evidenced by findings that IL-33-deficient mice exhibit reduced responses to LPS^1,44,45^. That this is not the case for LPS-induced IL-13 responses in BMMCs suggests an unexpected requirement for ST2 in TLR4 signalling or alternatively that ST2 is required for downstream signalling driven by an autocrine factor released in response to LPS. Such a putative autocrine factor is unknown but does not appear to be IL-33 as although TLR-2 or −9 signalling can induce IL-33 expression in BMMCs^46^, this is not the case for LPS/TLR4^32,37^.

It is worth bearing in mind, however, that IL-33 can recruit additional receptors such as SIGIRR, which acts to negatively regulate TLR responses^17^ and has been shown to suppress the Type-2 inflammation-promoting actions of IL-33/ST2 signalling^47^, but can be downregulated by LPS in monocytes and neutrophils^48^. IL-33 has also been shown to induce formation of ST2, IL-1RAcP and c-Kit complexes and indeed, the presence of the c-kit ligand, stem cell factor is required for mast cell cytokine responses^49^. Likewise, the vesicular transport protein, TMED-1 has been reported to be required for optimal IL-33 induction of cytokine production by HEK-293 cells and HUVECs^50^. Differential expression or recruitment of such regulatory elements may therefore be involved in shaping the precise IL-33 functional responses delivered depending on the mast cell subset and inflammatory context.

In any case, MyD88 provides a potentially important point of convergence in driving type-2 cytokine production in response to crosstalk amongst ST2, TLR4 and FcεRI receptors: this is because in addition to the widely established role of MyD88 in ST2 and TLR4 signalling, LPS has recently been shown to stimulate IL-13 release from mast cells via a MyD88-BLT2 signalling pathway that has previously been implicated in FcεRI signalling^5^. Indeed, whilst FcεRI crosslinking upregulates expression of BLT2, a LTB4 receptor on BMMCs, blockade with an antagonist or siRNA abolishes FcεRI production of type-2 cytokines^19^ and also the enhanced responses resulting from costimulation via LPS/TLR4 and FcεRI^5^. Moreover, antisense blockade of BLT2 strongly reduces airway inflammation and AHR in an OVA-induced IgE-dependent mouse model of asthma^20^ and a BLT2 antagonist suppresses the strongly enhanced levels IL-13 observed in this model following administration of LPS^5^. Thus, as ES-62 targets aberrant MyD88 signalling (reviewed^15,16,42^), this MyD88-BLT2 pathway potentially provides a unifying mechanism for its ability to suppress FcεRI and LPS/TLR4-stimulated cytokine responses in PDMCs and BMMCs^13,14^, as well as those modulated by IL-33/ST2, particularly as ES-62 does not modulate expression of either FcεRI or ST2 in PDMCs^13^.

The clinical relevance of our finding that ES-62 can target the ST2-FcεRI-TLR4 crosstalk driving allergic inflammation is not yet clear. However, we have previously shown ES-62 to be protective in mouse models of acute and chronic asthma and also contact hypersensitivity, and to induce hyporesponsiveness of mast cells *in vivo*, which persists even following *ex vivo* challenge with allergen or LPS^10,12,51^. Although, serosal (PDMC) and mucosal (BMMC)-like mast cells have been reported to differentially display strong degranulation versus cytokine/chemokine responses respectively, the precise roles of distinct mast cell phenotypes in pathogenesis, resolution of inflammation and tissue repair in allergy are not yet clear. This uncertainty reflects that mast cells only fully mature functionally, and in a bespoke manner, in response to tissue-specific factors and the inflammatory context of their microenvironment, as evidenced by the ability of BMMCs to reconstitute both serosal and mucosal mast cells *in vivo* (reviewed^52^). Nevertheless, ES-62 appears able to reduce mast cell numbers and suppress their proinflammatory functions in bronchoalveolar lavage fluid (BALF; mucosal), airway epithelial lung tissue and skin (serosal/connective tissue) to reset immune homeostasis in models of allergic inflammation^10,12,51^ as well as differentially target both mature mast cells and their immature precursors in a mast cell subtype-, receptor and response-specific manner *in vitro*^13^. Collectively, these findings suggest that the parasite product is capable of distinct actions reflecting the plasticity of the mast cell population and site and inflammatory context of the microenvironment in order to effect resolution of harmful inflammation and promote tissue repair whilst maintaining protective responses to fight infection. Consistent with this idea that ES-62 offers “safe” immunomodulation^16,42^, while it typically reduces degranulation and cytokine response of “healthy“ mast cells *in vitro* by some 20–30%, it exhibits more profound inhibition of mast cell responses under conditions of allergic inflammation^10,12,51^.

Reflecting potential differential effects, our preliminary data (Coltherd, Harnett & Harnett, unpublished) indicate that the protection afforded against acute OVA/alum-induced AHR is associated with significant reduction in the levels of mast cell protease (MCPT)-1 in the BALF, that presumably reflects ES-62-mediated suppression of mast cell degranulation. By contrast, in the chronic OVA (non-alum) model of asthma, where mast cells play roles both in the early inflammatory phase (via degranulation) and also in the later airway remodelling phases of disease, whilst reduced levels of mast cells were found in the lungs of ES-62-treated mice^12^, this was not associated with a significant decrease in BALF levels of MCPT-1 in the chronic phase of disease. Likewise, and consistent with release of IL-33 being very rapid and transient in nature in models of asthma (reviewed^53^), we did not detect any differences in BALF levels of IL-33 amongst the experimental groups in either the acute or chronic models of asthma. However, we did find that the BALF levels of soluble ST2, a mediator which can inhibit LPS-induced shock *in vivo*^54^, were significantly reduced in OVA, but not OVA + ES-62 mice, relative to those in the healthy PBS-treated control mice in the chronic asthma model.

Thus, we addressed how ES-62 might impact on IL-33/ST2 signalling in asthma by investigating its effects in a chronic OVA/alum model of asthma in WT and ST2 KO BALB/c mice^55^: whilst the majority (4/6) of the WT mice with OVA-induced pathology died before the end of the protocol, all such mice (6/6) treated with ES-62 survived. Likewise, all of the ST2 KO mice, irregardless of whether they were in the OVA or OVA + ES-62 cohorts, survived. Such protection by ES-62 and/or ST2 deficiency was associated with reduced serum levels of OVA-specific IgE with no significant differences in OVA-specific IgE levels being detected amongst the OVA + ES-62 WT, OVA ST2 KO and OVA + ES-62 ST2 KO mice. Allergen-specific IgE is pathogenic in asthma, presumably by driving FcεRI-mediated degranulation and cytokine secretion, including autocrine release of IL-33 and downstream effector signalling: thus, collectively these data suggest that at least some of the *in vivo* protective effects of ES-62 in chronic models of asthma reflect modulation of IL-33/ST2 signalling. Whilst IL-33 promotes FcεRI and TLR4 cytokine responses, particularly at low levels of allergen, and has been shown at the single cell level to boost FcεRI-mediated degranulation and generate high responder mast cells (reviewed^56^), low sub-activating levels of IL-33 can induce ST2-dependent BMMC hyporesponsiveness to bacterial cell wall (TLR4-agonist) products but not allergens, *in vitro*^31^. Such IL-33-mediated hyporesponsiveness could provide a homeostatic mechanism to limit undue inflammation and tissue damage at sites of microbial invasion^31^. Thus, ES-62-mediated suppression of FcεRI-mediated release of IL-33 and/or consequent ST2-promotion of FcεRI and TLR4 responses may effectively harness this homeostatic mechanism for maintaining mast cell unresponsiveness and preventing allergic inflammation.

## Materials and Methods

### Mice

ST2-deficient mice and their wild type (WT) littermates were bred in-house on the BALB/c background and maintained at the University of Glasgow as we described previously^43^. Additional BALB/c and C57BL/6 mice were obtained from Harlan-Olac (now Envigo): we observed variability in the signalling and levels of cytokine production by mast cells derived from the different strains of mice and also between BALB/c mice obtained from commercial sources and those WT littermates bred in house^13,14^. All animals were specified pathogen-free and maintained under standard *ad libitum* conditions in accordance with Home Office, U.K. animal guidelines and with the approval of the Animal Welfare Ethical Review Board (AWERB) at the University of Glasgow and the Ethical Review Board of the University of Strathclyde,

### Preparation of endotoxin-free ES-62

ES-62 was purified to homogeneity (as confirmed by SDS-PAGE) from conditioned medium of cultures of adult *Acanthocheilonema viteae* using endotoxin-free reagents and used at < 0.003 endotoxin units/ml as determined using a Limulus Amebocyte Lysate QCL-1000 kit (Lonza Biologics)^57^.

### Peritoneal-derived mast cells (PDMCs) and bone marrow–derived mast cells (BMMCs)

Mature serosal mast cells, as characterised by their size, morphology and expression of proteoglycans like heparin (as indicated by toluidine blue staining) and tryptase (by enzyme histochemistry [Z-Gly-Pro-Arg-MNA as substrate and Fast Garnet as chromogen]) were isolated from the peritoneal cavity (Supplementary Figure 1) and such populations of PDMCs were enriched as described previously^13,58,59^. Briefly, non-adherent cells harvested from the peritoneal cavity were cultured at 0.3 × 10^6^ cells/ml in complete RPMI medium (RPMI medium with 10% FBS, 4 mM L-glutamine, 100 U/ml penicillin, 100 μg/ml streptomycin, 1 mM sodium pyruvate, 100 μM non-essential amino acids and 50 μM β-mercaptoethanol; Invitrogen Life Technologies) supplemented with 4% conditioned medium from the SCF-secreting CHO cell line, KLS-C^13^.

BMMCs were derived by culture (at 0.5 × 10^6^ cells/ml) of single cell suspensions of BM cells from femurs and tibias in complete RPMI medium supplemented with conditioned medium from KLS-C (1%; SCF) and IL-3-producing TOP3 (3%; IL-3) cell lines. These conditions generate cells, which although phenotypically similar to immature mast cells are widely used as a model of mucosal mast cells^13,58,59^

PDMCs and BMMCs were cultured at 37 °C/5% CO_2_ in tissue culture-treated flasks (Greiner Bio-one) for at least 28 days, with adherent cells being discarded. A purity of > 95% mast cells was routinely obtained as evidenced by flow cytometric analysis of surface expression of CD117, FcεR1 and ST2 and histological analysis of toluidine blue staining^13^. Briefly, following blocking of Fcγreceptors with anti-CD16/32 antibodies (clone 2.4G2 hybridoma supernatant), cells were labelled with the relevant flurochrome-conjugated or biotinylated antibodies (antibodies specific for CD117 [#47–1171], FcεRI [#11–5898, 12–5898] and TLR4/MD2 [#12–9924, 17–9924] were obtained from eBioscience; whilst that reactive against ST2, was obtained from MD Bioproducts [#101001 F]) and developed where appropriate with flurochrome-conjugated streptavidin. Dead cells were excluded from the analyses by use of the Live/Dead® Viability/Cytotoxicity Kit (Invitrogen) or staining with 7-AAD (7-Amino Actinomycin D; eBioscience), the latter immediately prior to acquiring a minimum of 10,000 data events (Becton Dickinson LSR II or FACSCalibur™ flow cytometer), and expression levels subsequently analysed using FlowJo software (Tree Star Inc). Gating strategies were as reported previously^13^.

### Mast Cell Stimulation

Mast cells were sensitised with murine anti-DNP IgE (0.5 µg/ml; IgE) in the presence or absence of ES-62 (2 µg/ml) for 18 h prior to stimulation, unless otherwise specified. Cells were incubated (generally 10^6^ cells/ml) with medium alone (as a negative control), IL-33 (10 ng/ml) and/or DNP-HSA (0.5 µg/ml) to crosslink FcεR1 (XL), LPS to activate TLR4 (0.5 µg/ml; *Salmonella minnesota*) or phorbol myristate acetate plus ionomycin (both 1 µM; PMA + Iono) and supernatants assayed for cytokine release whilst the cell pellets prepared for Western Blot analysis were stored at −20 °C^13^. ELISAs for IL-6, IL-13 and CCL2 [MCP-1] (limits of detection 4 pg/ml, 4 pg/ml and 15 pg/ml respectively; eBioscience) were performed on triplicate samples and developed using TMB substrate and absorbances determined using a TECAN Sunrise Microplate reader^13^.

### Calcium Mobilisation

As described previously^13^, cells were loaded with Fura-2/AM (5 µM; Invitrogen) in HBSS medium (145 mM NaCl, 5 mM KCl, 1 mM MgSO_4_, 1 mM CaCl_2_, 10 mM HEPES) and supplemented with 0.18% (w/v) D-glucose and 0.2% (w/v) BSA for 30 min at 37 °C in the dark. Calcium-free HBSS supplemented with 100 µM EGTA (ethylene glycol tetra-acetic acid) was used for measurement of intracellular calcium mobilisation in the absence of extracellular calcium. Cells (10^6^) were stimulated as indicated at t = 50 s and measurements acquired in a Hitachi F-700 fluorescence spectrophotometer for up to 180 s, with calcium levels detected every 500 ms using excitation-emission ratios of 340/380 nm. Following each experiment Rmax and Rmin values were determined by the addition of 1% Triton-X100 and subsequent addition of 20 mM EGTA pH 7.4, respectively^13^.

### Mast Cell Degranulation

Detection of β-hexosaminidase release is widely used as a surrogate for histamine degranulation. Mast cells (0.2 × 10^6^) were suspended in 200 µL Tyrodes buffer supplemented with 1% FCS and stimulated as indicated for 30 min at 37 °C. Supernatants were assayed using a Tecan Sunrise microplate reader (at 405 nm), for release of β-hexosaminidase (normalised to the total cellular β-hexosaminidase determined following cell lysis by addition of 1% Triton-X 100) by incubation with 1 mM *p*-nitrophenyl-*N*-acetyl-β-d-glucosamine in 0.05 M citrate buffer, pH 4.5, followed by quenching with 0.1 M sodium bicarbonate buffer as described previously^13^.

### Western Blotting

Mast cells (2 × 10^6^/ml) were lysed by the addition of 50 µL ice-cold, modified RIPA lysis buffer (50 mM Tris buffer, pH 7.4 containing 150 mM sodium chloride, 2% (v/v) NP40, 0.25% (w/v) sodium deoxycholate, 1 mM EGTA, 10 mM sodium orthovanadate, 0.5 mM phenylmethylsulfonylfluoride and chymostatin, leupeptin, antipain and pepstatin A [all at 10 µg/ml]) and resulting cell lysates stored at −20 °C as described previously^13^.

Cell lysates (30–40 µg protein per lane; BCA protein assay, Thermo Pierce) were resolved using the XCell *SureLock* Mini-Cell kit with NuPAGE Novex high-performance pre-cast Bis-Tris gels and NuPAGE buffers and reagents (Invitrogen) followed by transfer to membranes and Western Blotting as described previously^13^. Briefly, following blocking for 1 h in TBS/Tween-20/5% non-fat milk, membranes were incubated overnight at 4 °C with the appropriate primary detection antibody in TBS/Tween-20 with either 5% non-fat milk or 5% BSA prior to exposure to the appropriate horseradish peroxidise (HRP)-conjugated secondary antibody for 2 h at room temperature and visualisation using the ECL detection system and Kodak X-Ray film. The data presented in the main text figures are cropped blots but representative full-length images of the relevant antibody specificities are shown in Supplementary Figure 1.

### Statistical Analysis

Statistical analysis was performed using Graphpad Prism (Graphpad Software Inc) using unpaired t-tests or Mann-Whitney analysis for non-parametric data, two-way ANOVA or one-way ANOVA with Bonferroni’s post-test where p is significant at *p < 0.05, **p < 0.01 or ***p < 0.001.

### Data availability

No meta data sets were generated or analysed during the current study and so all data generated or analysed during this study are included in this article and associated Supplementary file.

### ST2-deficient mice

The authors would also like to give special thanks to Dr Andrew McKenzie (Medical Research Council Laboratory of Molecular Biology, Cambridge) for originally providing the ST2 KO mice to the University of Glasgow and which we used and described previously^43^.

## Electronic supplementary material

Supplementary Information
