# Supplementary material for: IL-33/ST2 signalling and crosstalk with FcεRI and TLR4 is targeted by the parasitic worm product, ES-62
Source: Sci Rep. 2018 Mar 14;8:4497. doi: 10.1038/s41598-018-22716-9 (PMC5852134; doi:10.1038/s41598-018-22716-9)
Supplement: Supplementary file 1 — Supplementary Information [file 41598_2018_22716_MOESM1_ESM.pdf]

IL-33/ST2 signalling and crosstalk with FcεRI and TLR4 is targeted by the parasitic worm product, ES-62

Dimity H Ball[1], Lamyaa Al-Riyami[2], William Harnett[2] and Margaret M Harnett[1]

[1]Institute of Infection, Immunity and Inflammation, College of Medical, Veterinary and Life Sciences, University of Glasgow, Glasgow G12 8TA and [2]Strathclyde Institute of Pharmacy and Biomedical Sciences, University of Strathclyde, Glasgow G4 0RE

Corresponding author: Margaret M Harnett

Institute of Infection, Immunity and Inflammation,

College of Medical, Veterinary and Life Sciences

University of Glasgow

Glasgow G12 8TA

Tel: 0141-330-8413

E-mail: Margaret.Harnett@glasgow.ac.uk

Supplementary Figure 1 Histological characterisation of PDMCs and representative full-length images of the Western Blot analysis of BMMCs and PDMCs shown in Figs. 2, 4 and 6. Panels **a** & **b**: following removal of adherent cells, cells from peritoneal washes of 10-week old BALB/c mice were stained for proteoglycan (heparin) with toluidine blue (**a**) or for tryptase activity (**b**) and images obtained using an Olympus BX41TF microscope (scale bars 100 μm). In panels **c-e**, analysis of expression of IL-33 stimulated ERK (**c**, dually phosphorylated, activated pp42/pp44 ERK; **d**, total p42/p44 ERK) and NF-κB (**e**, IκB) activation in BMMCs (data shown in Fig. 2). Expression of the loading control, β-actin is shown in **f**; the residual staining of IκB following stripping and reprobing with anti-β-actin is evident. In panels **g** & **h**, analysis of expression of FcεRI stimulated ERK (**g**, pp42/pp44; **h**, p42/p44) activation in PDMCs (Fig. 4) and in panels, **i & j**, analysis of expression of LPS stimulated ERK (**i**, pp42/pp44; **j**, p42/p44) activation in BMMCs (Fig. 6).

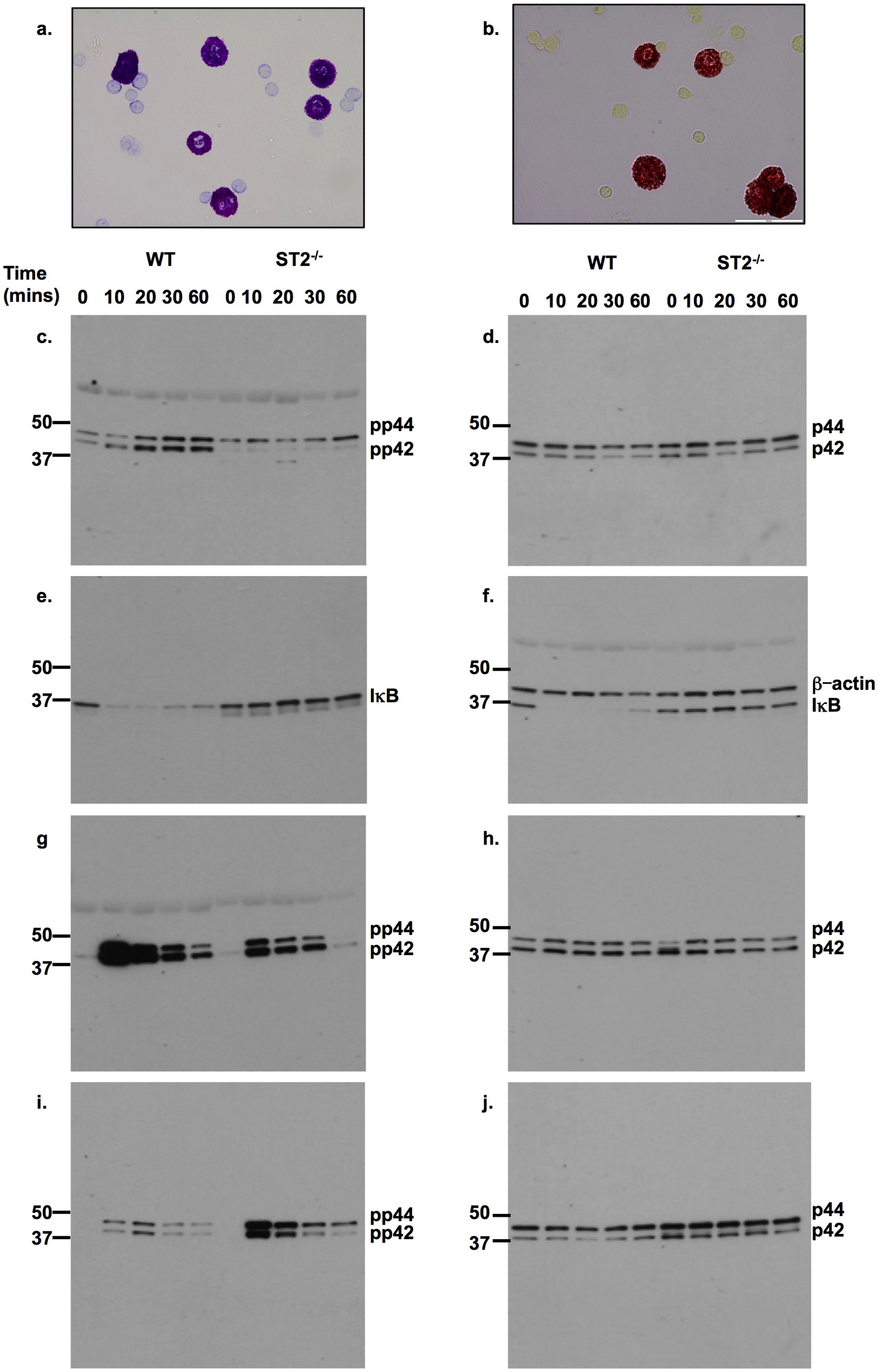
a.
b.
WT
ST2-/-
Time (mins)
0 10 20 30 60 0 10 20 30 60
c.
d.
50
37
pp44
pp42
p44
p42
e.
f.
IκB
β-actin
g
h.
i.
j.
